# Biological Properties of Latex, Aqueous Extracts and Bee Products of *Euphorbia officinarum* L.: A Short Review

**DOI:** 10.3390/molecules27217200

**Published:** 2022-10-24

**Authors:** Oumaima Boutoub, Lahsen El Ghadraoui, Maria Graça Miguel

**Affiliations:** 1 Faculdade de Ciências e Tecnologia, Campus de Gambelas, Universidade do Algarve, 8005-139 Faro, Portugal; 2 Laboratory of Functional Ecology and Environment, Faculty of Science and Technology, University Sidi Mohamed Ben Abdallah, BP 2202, Fez 20000, Morocco; 3 Mediterranean Institute for Agriculture, Environment and Development, Campus de Gambelas, Universidade do Algarve, 8005-139 Faro, Portugal

**Keywords:** *Euphorbia officinarum*, spurge, triterpene, triterpene derivatives, ingol diterpenes, insecticidal activity, antimicrobial activity, antioxidant activity, α-glucosidase inhibitory activity

## Abstract

*Euphorbia officinarum* L. is a Moroccan endemic plant known as “Tikiout” and “Daghmus” that can also be found in Mauritania, Western Sahara, and Algeria. In the present review, “*Euphorbia officinarum*”, “metabolites” “hemisynthesis” were the keywords used for the research in the Web search engine Google Scholar and in the database Web of Science. Triterpenes, phytosterols and ingol diterpenes were isolated and identified in the latex of Moroccan *E. officinarum*. More than sixty triterpenes were obtained by hemisynthesis from natural triterpenes. Some of these derivatives had insecticidal and antimicrobial activity (phytopathogenic bacteria). The total phenol content and the antioxidant and anti-α-glucosidase activities were dependent on the time and temperature of extractions and also on the plant solvent ratio. The antioxidant activity of monofloral honey of *E. officinarum* origin was attributed to the phenol fraction (this fraction, previously isolated from honey samples, had better activity than the entire honey).

## 1. Introduction

**Figure 1:**
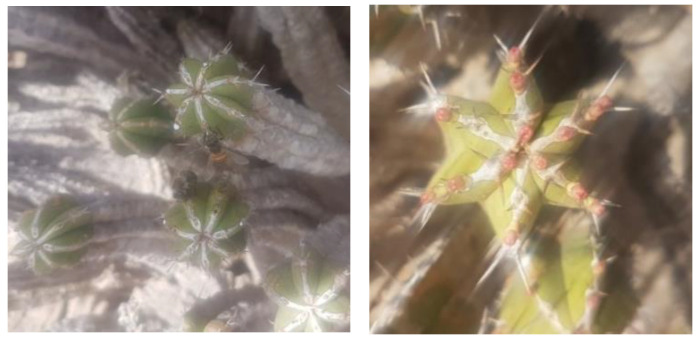
*Euphorbia officinarum* L. in Moroccan fields.

**Figure 2:**
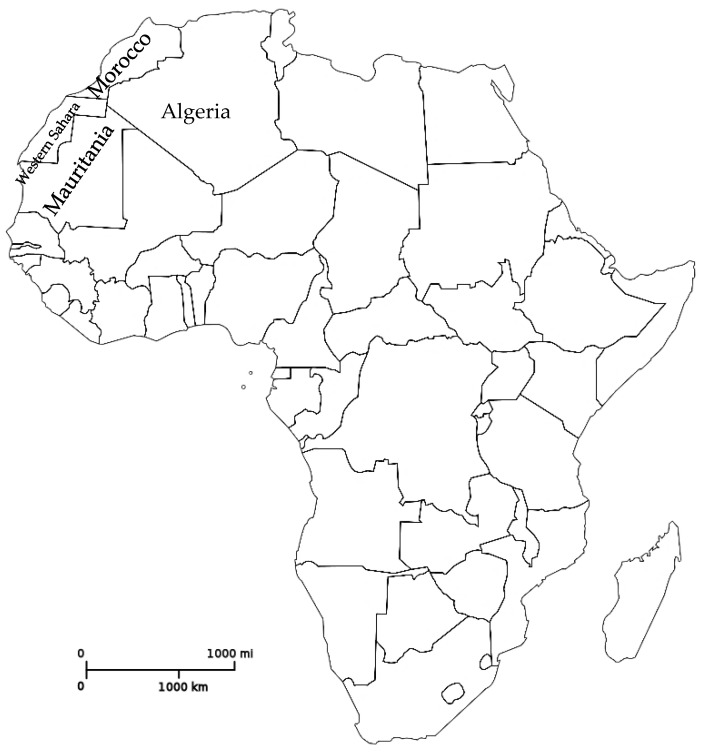
Geographical location of *Euphorbia officinarum*. Adapted from https://en.wikipedia.org/wiki/File:Blank_Map-Africa.svg (accessed 22 October 2022).

*Euphorbiaceae* family encloses 6000 species of plants, and genus *Euphorbia* is the largest in the spurge family. *Euphorbia* are succulent plants that may be found from Africa to the Canary Islands, in Madagascar, India, and the Americas and even Australia [1]. *Euphorbia officinarum* L. (Figure 1) is a Moroccan endemic plant known as “Tikiout” and “Daghmus”. This species can also be found in Mauritania, Western Sahara, and Algeria [1] (Figure 2). *E. officinarum* belonging to the *Euphorbia* genus has the presence of a milky white latex, which has been studied not only for its chemical characterization but also due to its biological properties [2]. Generally, this latex has been used since ancient times in folk medicine although containing harmful compounds. The term *Euphorbia* was named in honour of “Euphorbus”, the physician of King Juba II of Mauritania, who paid attention to the medicinal properties of *E. officinarum*, for the first time [3]. There is one infra-specific taxon of the species *Euphorbia officinarum* L. (*E. officinarum* subsp. *echinus* (Hook.f. & Coss.) Vindt) [4].

Since ancient times, *E. officinarum* has been used in folk medicine, although currently it has been determined that this is obsolete, at least for some ailments. For example, there are still descriptions that the milky sap of this species is used in earache and as emetic in Buxar district, India [5], although this utilization as emetic has already been considered outdated [6]. Anti-diabetic utilization of *E. officinarum* is particularly cited in diverse places in Morocco and almost always under powder obtained from aerial parts of *E. officinarum* subsp. *echinus*. Some local examples of its use include Agadir Ida Outanane region (Southwest Morocco) [7], Tata Province [8], Chtouka and Tiznit (western Anti-Atlas) [9], multiple regions [10] and under decoction in Beni Mellal-Khenifra [11]. *E. officinarum* L. is also used as anti-diabetic, according to different authors [10,11,12,13].

*E. officinarum* in a mixture with other plants (*Opuntia ficus-barbarica*, *Zea mays* and *Ziziphus lotus*) and honey has been used in the treatment of pyelonephritis and cystitis, in Moroccan Sahara [14]; the flowers and roots of *Euphorbia officinarum* subsp. *echinus* are also reported in the treatment of pyelonephritis in Central Morocco [15]. *E. officinarum* subsp. *echinus* has also been reported in the treatment of wounds, skin infections and abscesses in diverse places in Morocco [16,17,18] and by the Sahrawi refugees in Algerian refugee camps [19]. Although Idm’hand et al. [18] had noted the utilization of that species in the treatment of skin diseases, their work indicated that the specific treatment purpose of *E. officinarum* subsp. *echinus* was the elimination of helminths.

In Morocco, the root powder of *E. officinarum* subsp. *echinus* has also been reported in the treatment of cancer, although not specifying which type [16,20,21]. Through ethno-botanical interviews and vegetation surveys in a Saharan Moroccan village, Blanco and Carrière [22] reported that *E. officinarum* subsp. *echinus* use presented a Smith Salience index:

(1) $$\mathrm{Smith}\;\mathrm{Salience}\;\mathrm{index}=\frac{\sum_{i=1}^{N}\frac{L_{i}-R_{a}+1}{L_{i}}}{N}$$

*N*: total number of informants; *L_i_*: size of the list for the informant *i*; *R_a_*: rank of appearance of the ethnospecies and frequency of 0.239 and 38%, respectively. Other uses have also been described for *E. officinarum*, such as for respiratory and circulatory systems [23], and as gum-resin for headache, paralysis and apoplexy [24]. In Morocco, *E. officinarum* subsp. *echinus* aerial parts may be chopped and cooked as a vegetable salad [25,26], despite its relative high toxicity [2].

## 2. Secondary Metabolites Isolated from *Euphorbia officinarum* L. of Morocco

**Figure 3:**
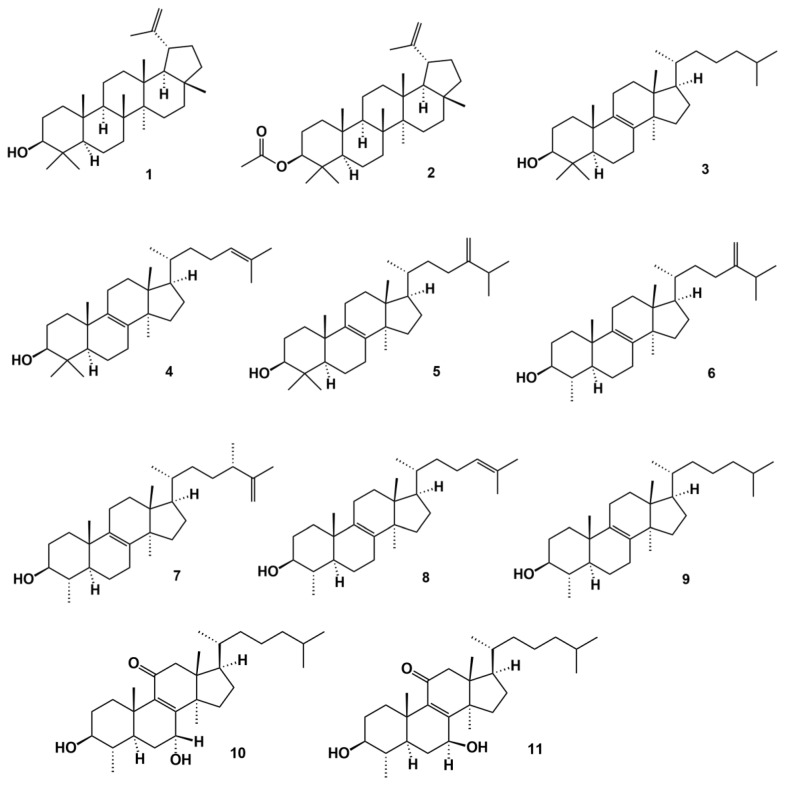
Triterpenic (**1** and **2**) and steroid compounds isolated and identified in the Moroccan latex of *Euphorbia officinarum*.

In 1985, for the first time, Ben Harref and Lavergne [27] isolated from the methanolic extract of the latex of *E. officinarum*, collected in the North Atlantic coast of Agadir (Morocco), nine compounds with triterpenic (lupeol (**1**), lupeol acetate (**2**)) and steroidal skeleton (lanostenol (**3**), lanosterol (**4**), 24-methylene lanostenol (**5**), 4α,14α-dimethyl-24-methylen-5α-cholest-8-en-3β-ol or obtusifoliol (**6**), 24(*R*)-4α,14α,24-trimethyl-5α-cholesta-8,25-dien-3-β-ol (**7**), 4α,14α-dimethyl-5α-cholest-8,24-dien-3β-ol (**8**), and 4α,14α-dimethyl-5 α-cholest-8-en-3-β-ol (**9**)) (Figure 2). In 1999, Daoubi et al. [28] isolated 3 steroidal compounds from a methanolic extract of *E. officinarum* latex collected in the North Atlantic coast of Agadir (Morocco), one of them being 4α,14α-dimethyl-5α-cholest-8-en-3-β-ol, already isolated and identified by Ben Harref and Lavergne [27] and two new ones described for the first time by the authors: 3β,7α-dihydroxy-4α,14α-dimethyl-5α-cholest-8-en-11-one (**10**) and 3β,7β-dihydroxy-4α,14α-dimethyl-5α-ergost-8-en-11-one (**11**) (Figure 3). The identification was established on the basis of proton-nuclear magnetic resonance (^1^H-NMR), carbon 13-nuclear magnetic resonance (^13^C-NMR), heteronuclear multiple bond correlation (HMBC), electron ionization mass spectroscopy (EIMS), and high-resolution electron ionization mass spectrometry (HREIMS).

**Figure 4:**
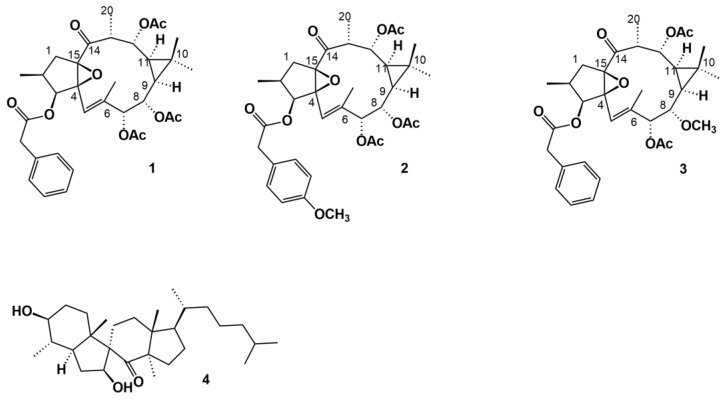
Phenylacetylingol derivatives (**1**, **2**, **3**) and spirotriterpenoid (**4**) isolated from the methanolic extract of *E. officinarum* latex.

Mazoir et al. [29] isolated 2 steroidal compounds, 4α,14α-dimethyl-24-methylen-5α-cholest-8-en-3β-ol or 4α,14α-dimethyl-5α-ergosta-8,24-dien-3β-ol or obtusifoliol (**6**) and 4α,14α-dimethyl-5α-cholest-8-en-3β-ol (**9**), previously identified by Ben Harref and Lavergne [27] from the latex of *E. officinarum* (Figure 3) from which they obtained derivatives, by oxidation of the hydroxyl group at C_3_ with chromic anhydride. Nevertheless, Mazoir et al. [29] reported 4α,14α-dimethyl-5α-cholest-8-en-3β-ol as being synonymous of 31-norlanosterol. However, we believe that the identified compound is in fact 4α,14α-dimethyl-5α-cholest-8-en-3β-ol because the chemical structure presented in the article is most definitely the one from this compound and not that of 31-norlanosterol. Beyond the triterpenic and steroidal compounds isolated from *E. officinarum*, Daoubi et al. [2] isolated and identified highly functionalized ingol diterpenes: ingol 7,8,12-triacetate 3-phenylacetate) (**1**), ingol 7,8,12-triacetate 3-(4-methoxyphenyl) acetate (**2**) and 8-methoxyingol 7,12-diacetate 3-phenylacetate (**3**) (Figure 4). Along with these three compounds, Daoubi et al. [2] also identified the novel spirotriterpene 3*S*,4*S*,5*R*,7*S*,9*R*,14*R*-3,7-dihydroxy-4,14-dimethyl-7[8→9]-abeo-cholestan-8-one (**4**) (Figure 4). The structure was established following the same procedures already described by the same team [28]. The plant origin was the same as previously reported [2,28]. The authors also proposed a possible biosynthetic pathway of the spirotriterpene compound from 4α,14α-dimethyl-5α-cholest-8-en-3-β-ol, since they coexist in the same plant. The hypothesis presented by the authors is based on other results observed for *E. supina* and *Ficus microcarpa*.

## 3. Biosynthesis of Diterpenes, Triterpenes and Sterols

**Figure 5:**
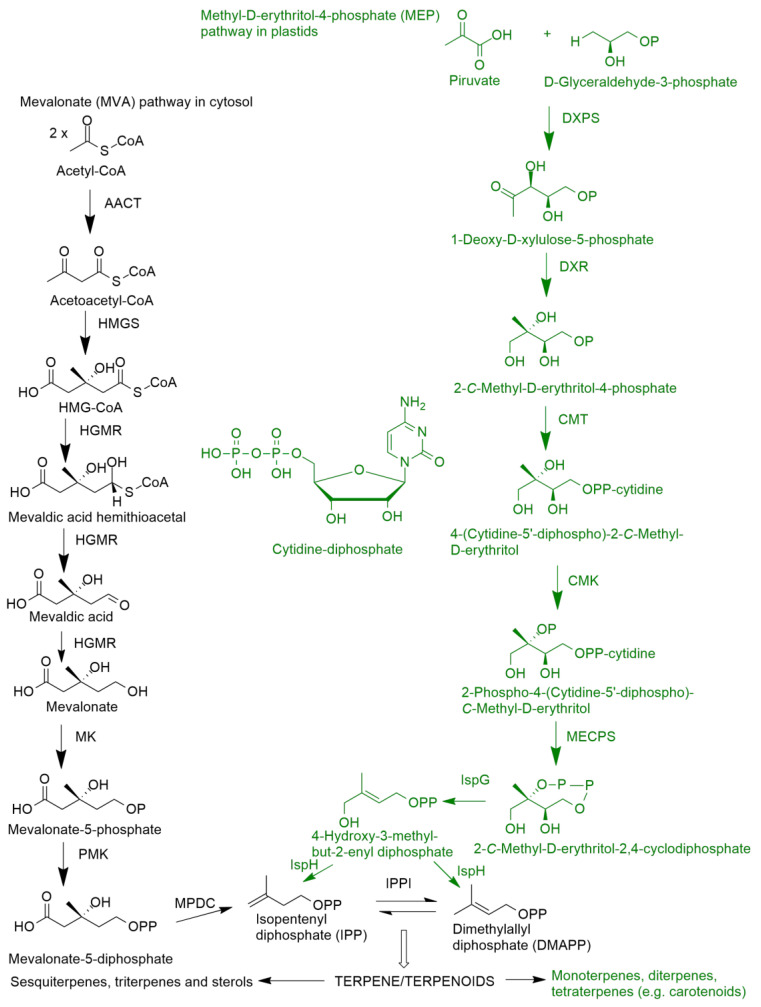
Biosynthesis of isopentenyl diphosphate (IPP) and dimethylallyl diphosphate (DMAPP) by the mevalonate (MVA) and methyl-D-erythritol-4-phosphate (MEP) pathways as well the enzymes involved in such pathways. AACT: Acetoacetyl-CoA-thiolase; CMK: 4-diphosphocytidyl-2-C-methyl-D-erythritol kinase; DXR: 1-deoxy-D-xylulose-5-phosphate reductase; DXPS: 1-deoxy-D-xylulose5-phosphate synthase; HDR: (E)-4-hydroxy-3-methyl-but-2-enyl diphosphate reductase; HMGR: 3-hydroxy-3-methylglutaryl-CoA reductase; HMGS: 3-hydroxy-3-methylglutaryl-CoA synthase; IPPI: isopentenyl diphosphate isomerase; IspG: 4-hydroxy-3-methylbut-2-enyl diphosphate synthase; IspH: 4-hydroxy-3-methylbut-2-enyl diphosphate reductase; MDPC: mevalonate-5-diphosphate decarboxylase; MECPS: MEP cytidyltransferase; MK: mevalonate kinase; PMK: phosphomevalonate kinase (adapted from [31]).

Terpenoids are assembled from C_5_ units (isoprene-like), and the number of repetitions of this unit, followed by cyclization reactions, rearrangements and oxidation of the terpene skeleton determine the huge diversity of structures (more than 80,000) [30]. Nevertheless and concerning the biosynthesis of these natural compounds, they have as central intermediates the isopentenyl diphosphate (IPP) and its isomer dimethylallyl diphosphate (DMAPP). Two distinct pathways generate these C_5_ precursors: the mevalonate pathway (MVA), which occurs in cytosol, and the deoxyxylulose-5-phosphate (DXP), more recently known as methyl-D-erythritol-4-phosphate (MEP), which occurs in plastids (Figure 5) [31]. The MVA pathway provides cytosolic metabolites, such as C_30_ triterpenes and their saponin derivatives, and C_27_-C_29_ steroids, plus some C_15_ sesquiterpenes, whereas MEP provides C_10_ monoterpenes, C_20_ diterpenes, C_40_ tetraterpenes (carotenoids), some C_15_ sesquiterpenes and the prenyl side chains of chlorophyll and plastoquinones [31]. These terpenes can then undergo oxygenation through the activity of cytochrome P_450_ monooxygenases (P_450_), followed by the introduction of other functional groups, originating more than 80,000 natural products [30].

**Figure 6:**
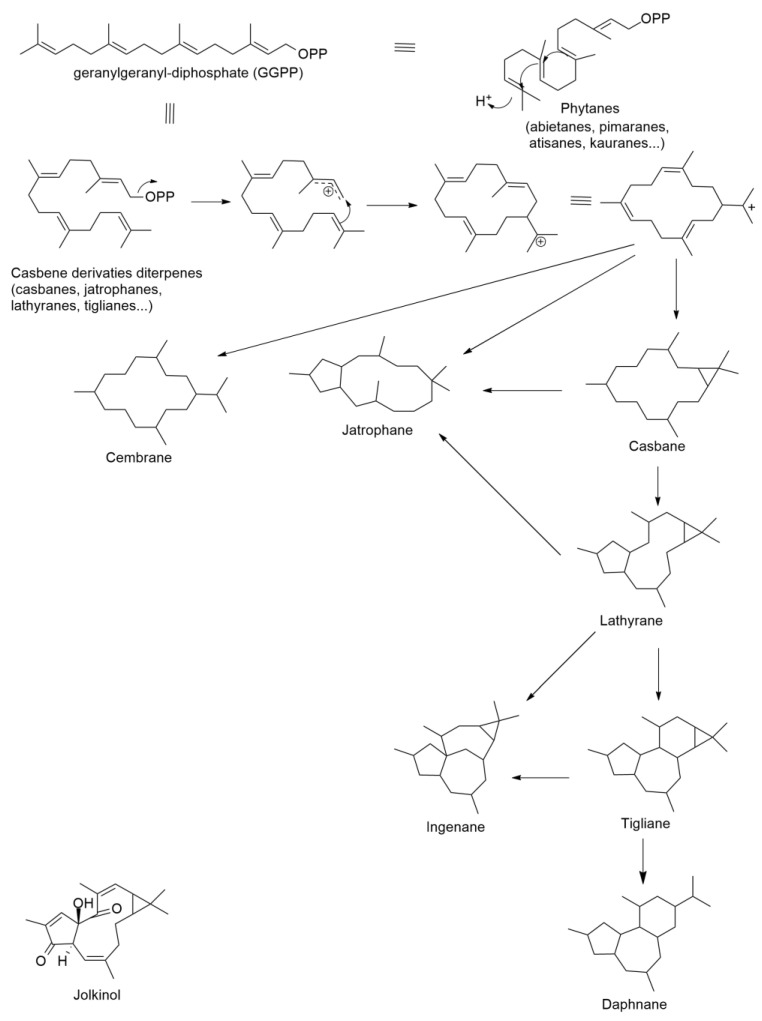
Schematic biogenesis of macrocyclic and polycyclic diterpenes derived from casbene (adapted from [32]).

Many studies have demonstrated that species of the family Euphorbiaceae are producers of unique diterpenoids, isolated from milky latices, which belong to the macrocylic diterpenes with diverse skeletons (jatrophane, lathyrane, terracinolide, ingenane, pepluane, paraliane, and segetane) (Figure 6) [32]. All skeletons have geranyl geranyl-diphosphate as precursor, as casbene is the precursor of the macrocyclic and polycyclic diterpenes (Figure 6) and then proceed through intermediates such as jolkinol C [32].

Ingol 7,8,12-triacetate 3-phenylacetate (**1**), ingol 7,8,12-triacetate 3-(4-methoxy-phenyl) acetate (**2**) and 8-methoxyingol 7,12-diacetate 3-phenylacetate (**3**) have the lathyrane skeleton, that is, tricyclic diterpenes with a 4/11/3-ring system, an epoxy functionality at C-_4_ and C-_15_ and a double bond between C-_5_ and C-_6_ (Figure 3) [33], which precursors are most likely the ones just mentioned. Further, those ingol diterpenoids underwent redox, etherification and esterification modifications of the 5- and 11-membered rings (Figure 3). The presence of the phenylacetyl group is also frequent in lathyrane diterpenoids [34].

Although the biological properties of those lathyrane diterpenes have not been evaluated, Vela et al. [34] in their review work noted that terpenes belonging to this group have biological properties with clinical potential (cytotoxicity, multidrug resistant reversal ability, antiviral and anti-inflammatory properties, and capability to induce neural progenitor cell proliferation or differentiation into neurons).

**Figure 7:**
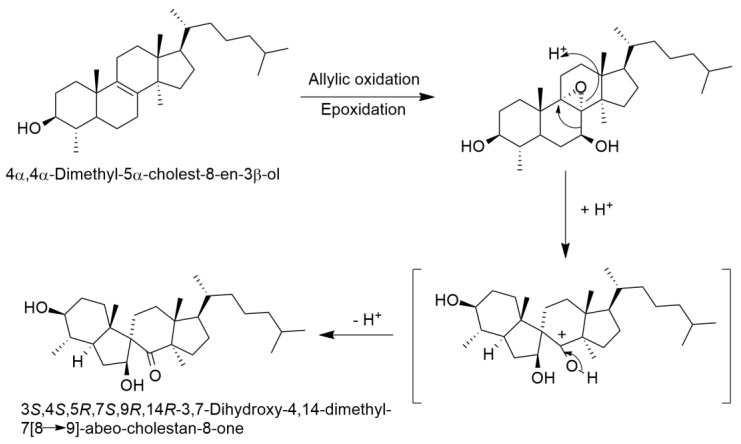
Hypothesis for the biosynthesis of the spirotriterpene (adapted from [2]).

As far as we know there are not any biosynthetic studies on the diterpenes, triterpenes and sterols in *Euphorbia officinarum*, only a hypothesis for the biosynthetic pathway of the spirotriterpene, 3*S*,4*S*,5*R*,7*S*,9*R*,14*R*-3,7-dihydroxy-4,14-dimethyl-7[8→9]-abeo-cholestan-8-one proposed by Daoubi et al. [2] (Figure 7).

**Figure 8:**
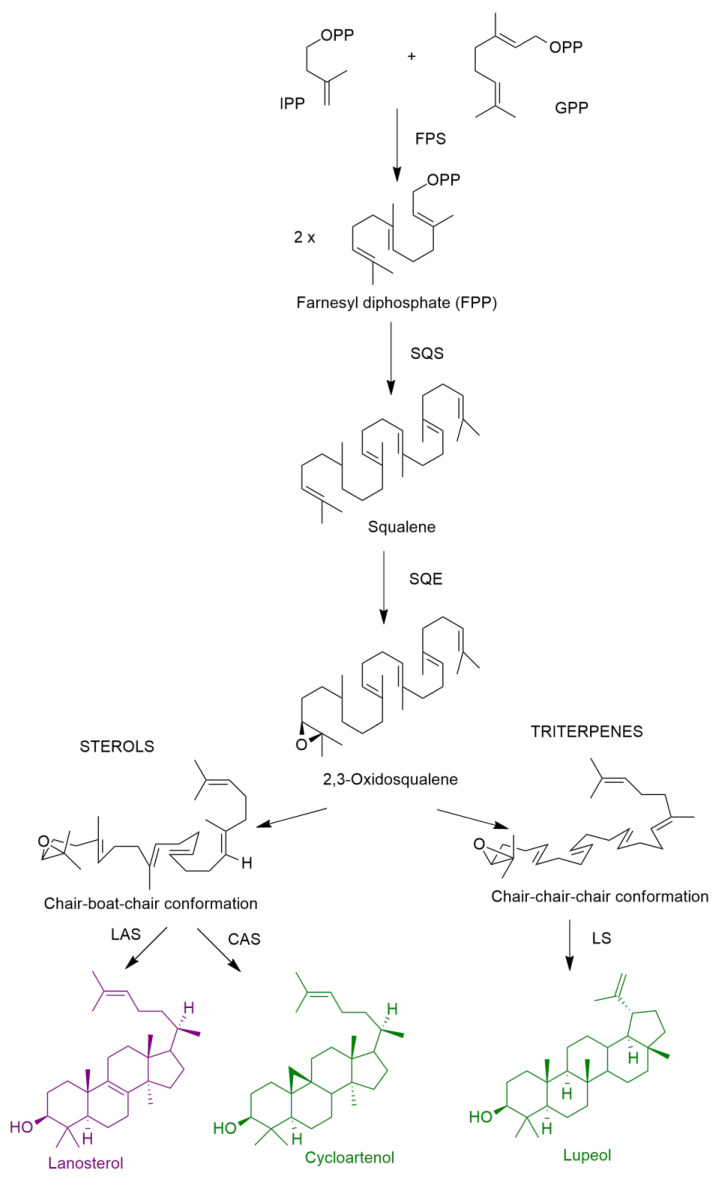
Schematic biosynthesis of sterols and triterpenes. CAS: cycloartenol synthase; FPS: farnesyl diphosphate synthase; GPP: geranyl diphosphate; LAS: lanosterol synthase; LS: lupeol synthase; IPP: isopentenyl diphosphate; SQE: squalene monooxygenase or epoxidase; SQS: squalene syntase (adapted from [35]).

Sterols and triterpenes are synthesized via the mevalonate pathway as aforementioned and the condensation tail-to-tail of two units of FPP originates the squalene (Figure 8). After the formation of this intermediate, there is the formation of 2,3-oxidosqualene, in animals, fungi and plants, which after cyclization leads to the sterols and triterpenes. In sterol biosynthesis, 2,3-oxidosqualene in the conformation chair-boat-chair is cyclized to lanosterol, in animal and fungi, whereas in plants, the cyclization originates cycloartenol (Figure 8) [35]. In the triterpenes, 2,3-oxidosqualene is folded into the chair-chair-chair conformation, which originates diverse structures such as lupeol, after cyclization, as depicted in Figure 8, a triterpenoid found in the *E. resinifera* latex [27].

**Figure 9:**
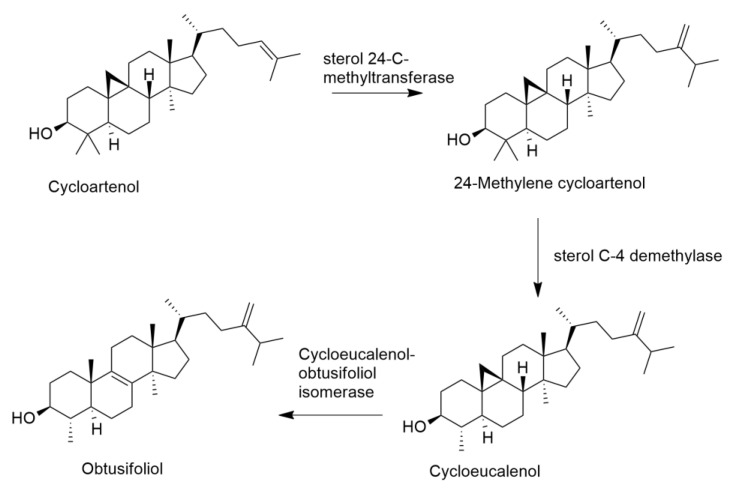
Schematic biosynthesis of obtusifoliol (adapted from [36]).

Obtusifoliol is a phytosterol, which means the presence of an extra one-carbon on the side-chain, attached at C_24_ and the substrate for alkylation is cycloartenol (Figure 9) [36]. Generally, the substrate for the biosynthesis of phytosterols is cycloartenol, whereas in fungi it is lanosterol [36]. However, in *E. lathyris*, Forestier et al. [37] reported lanosterol synthase in the cytoplasm of laticifers, in combination with cycloartenol synthase and butyrospermol synthases. Therefore, further studies are needed in order to better understand the role of cycloartenol and lanosterol in the biosynthesis of phytosterols in Euphorbiaceae.

## 4. Hemisynthesis of Triterpene Derivatives Isolated from *E. officinarum* Latex and Their Biological Properties

**Figure 10:**
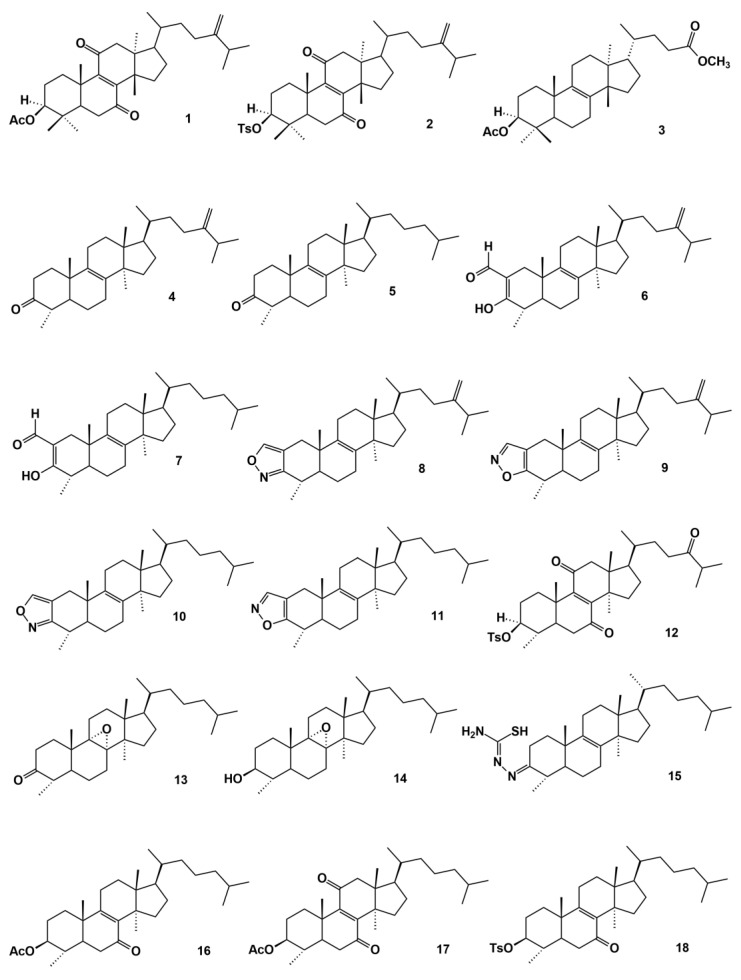

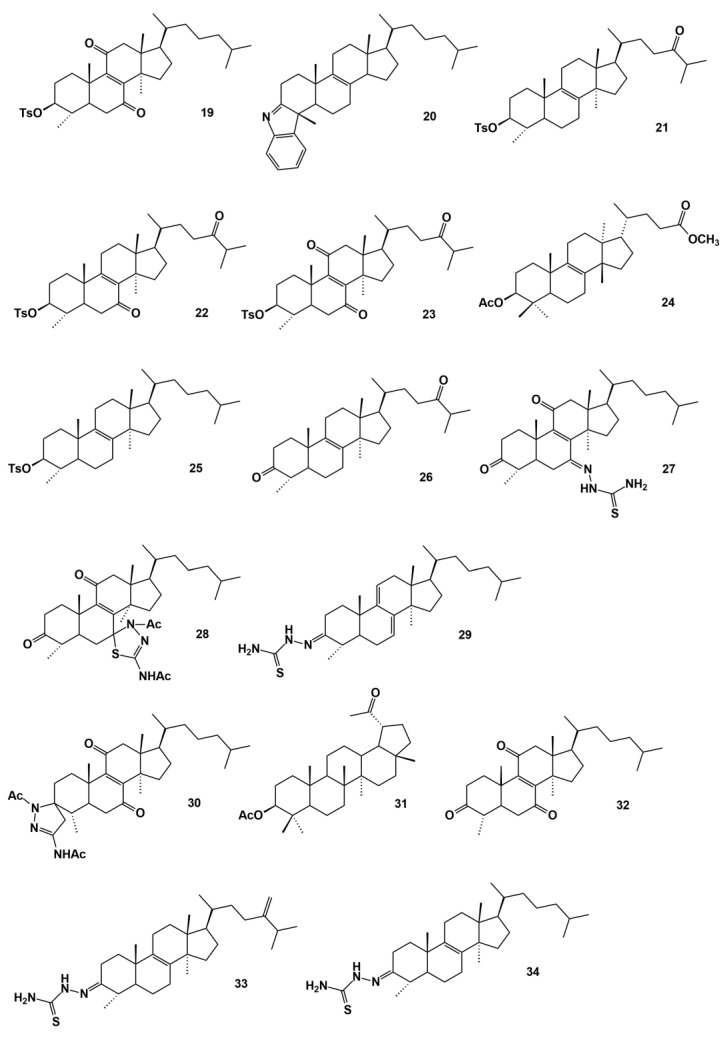

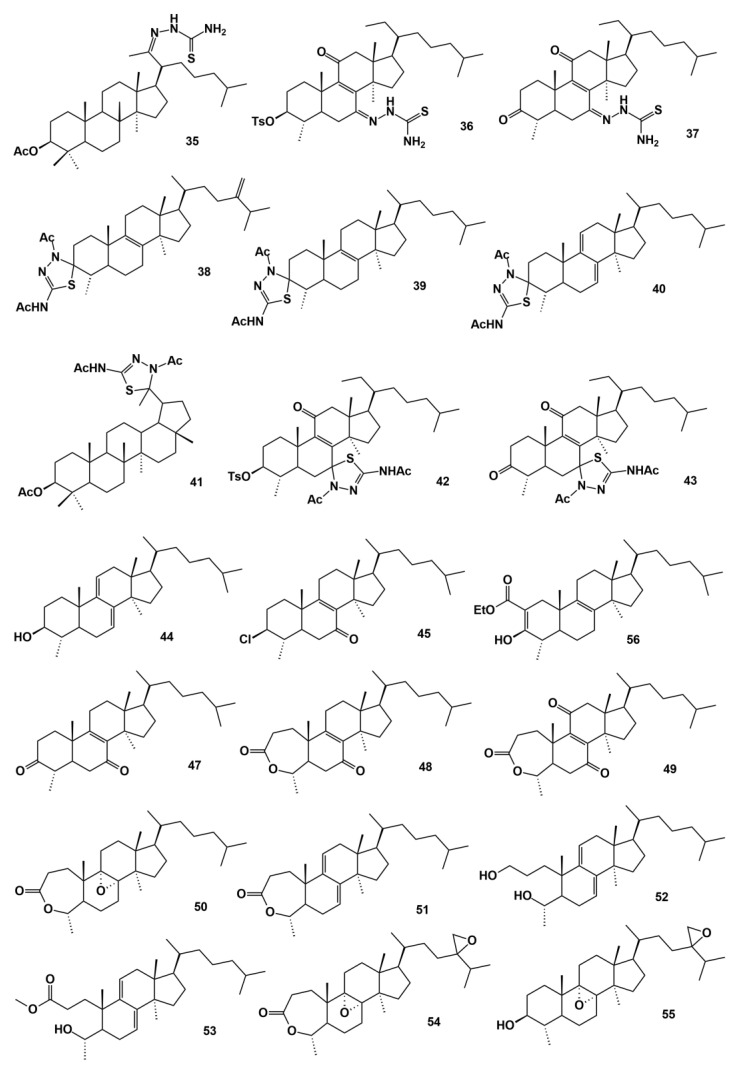

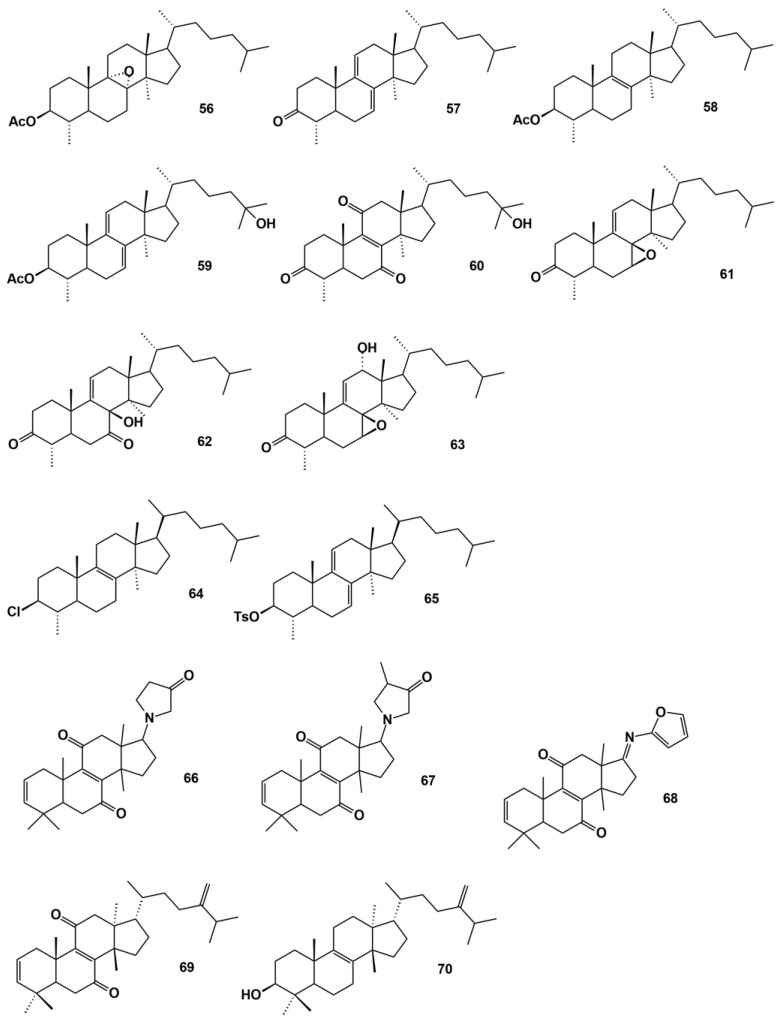
Triterpene derivatives obtained from natural triterpenes isolated from *E. officinarum* latex. Compounds **66**, **67** and **68** according to the structures presented in Figure 9 of Daoui et al. [48].

The biological properties found for latex samples or some of their isolated compounds (diterpenic, triterpenic or steroidal compounds) of *Euphorbia* species (cytotoxic, antimicrobial, human immunodeficiency virus type 1 reactivation, among others) [1,2,38,39,40,41], including *E. officinarum*, have led chemical modifications to obtain derivative compounds with the aim of improving the active properties. For example, Mazoir et al. (see below) published several works in order to obtain oxygenated triterpenic compounds that show good pharmacological activities. Generally, the procedures used were based on the oxidation of diverse triterpenic or steroidal compounds isolated from *E. officinarum*: from 24-methylene lanostenol to (3*S*)-acetyl-24-methyl-elemo-lanosta -8,24-diene-7,11-dione (**1**) (Figure 10), using chromic anhydride and acetone [42]; (3*S*)-tosyl-24-methyl-elemo-lanosta-8,24-diene-7,11-dione (**2**) from 3(*S*)-tosyl-24-methy-lene lanostenol using the same reagents [43]; (3*S*,5*S*,10*S*,13*S*,14*S*,17*S*)-methyl-3β-acetyl-25,26,27-trisnorlanost-8-en-24-oate (**3**) from eupho-lanosta-8,24-dien-3β-ol or lanosterol after oxidation by ruthenium(III) chloride trihydrate, followed by esterification and acetylation reactions [29]; 4α,14α-dimethyl-5α-ergost-8,24-dien-3-one (**4**) and 4α,14α-dimethyl-5α-cholest-8-en-3-one (**5**) from 4α,14α-dimethyl-5α-ergosta-8,24-dien-3β-ol and 4α,14α-dimethyl-5α-cholest-8-ene-3β-ol, respectively, with oxidation carried out using chromic anhydride and acetone; 2-formyl-4α,14α-dimethyl-5α-ergost-2,8,24-trien-3-ol (**6**) and 2-formyl-4α,14α-dimethyl-5α-cholesta-2,8-dien-3-ol (**7**) from the compounds (**4**) and (**5**), respectively, after treatment with ethyl formate, benzene and sodium methoxide [36]. From (**6**), Mazoir et al. [44] obtained [1,2]isoxazolo [4,3-b]-4α,14α-dimethyl-5α-ergosta-8,24-diene (**8**) after treatment of (**6**) with acetic acid and hydroxylamine hydrochloride, and [1,2]isoxazolo [4,5-b]-4α,14α-dimethyl-5α-ergosta-8,24-diene (**9**) after reaction of (**6**) with pyridine and hydroxylamine hydrochloride; and from 2-formyl-4α,14α-dimethyl-5α-cholesta-2,8-dien-3-ol (**7**), Mazoir et al. [44] obtained [1,2]isoxazolo [4,3-b]-4α,14α-dimethyl-5α-cholesta-8-ene (**10**) and [1,2]isoxazolo [4,5-b]-4α,14α-dimethyl-5α-cholest-8-ene (**11**) (Figure 10) using the same reagents for obtaining the compounds (**8**) and (**9**), respectively. Mazoir et al. [45] also obtained by hemisynthesis, from the derivative 3β-tosyl-5α-ergost-8,24-diene, the oxidized compound (3*S*,4*S*,5*S*,10*S*,13*R*,14*R*,17*R*)-4α,14α-dimethyl-3β-tosyl-5α-ergost-8-ene-7,11,24-trione (**12**) after reaction with ruthenium trichloride followed by allylic oxydation with chromic anhydride. The same authors [46] also obtained the derivative (4*S*,5*S*,10*S*,13*R*,14*R*,17*R*)-8α,9α-epoxy-4α,14α-dimethyl-5α-cholestan-3-one (**13**) (Figure 10), that is a triterpene functionalized with an oxirane bridge, from 4α,14α-dimethyl-5α-cholest-8-en-3β-ol, using the reagent chromic anhydride followed by epoxidation with a stoichiometric quantity of meta-chloroperbenzoic acid. This derivative could also be obtained from another derivative, 8α,9α-epoxy-4α,14α-dimethyl-5α-cholestan-3β-ol (**14**), after reaction with chromium anhydride [47].

The oxidation of 4α,14α-dimethyl-5α-cholest-8-en-3β-ol (**9**) with chromic anhydride produced the oxidized derivative 4α,14α-dimethyl-5α-cholest-8-en-3-one, which in the presence of thiosemicarbazide dissolved in ethanol and some drops of concentrated sulphuric acid produced the thiosemicarbazone derivative (**15**) [49]. From 4α,14α-dimethyl-5α-cholest-8-en-3β-ol (**9**), a major triterpene isolated from *E. officinarum* latex, it was also possible to obtain, by hemisynthesis, the derivatives 3β-acetoxy-4α,14α-dimethyl-5α-cholest-8-en-7-one (**16**) and 3β-acetoxy-4α,14α-dimethyl-5α-cholest-8-ene-7,11-dione (**17**), after acylation and oxidation processes [47]. The acylation can be replaced by a treatment with tosyl chloride followed by a similar oxidation condition, giving rise to the new derivatives 3β-tosyloxy-4α,14α-dimethyl-5α-cholest-8-en-7-one (**18**) and 3β-tosyloxy-4α,14α-dimethyl-5α-cholest-8-ene-7,11-dione (**19**) [47]. From the same steroidal compound isolated from *E. officinarum* latex, 4α,14α-dimethyl-5α-cholest-8-en-3β-ol, López-Rodríguez et al. [50] obtained by hemisynthesis 1-(1,5-dimethylhexyl)-3a,5b,12a,14a-tetramethyl-2,3,3a,4,5,5a,5b,11,12,13,14, 14a-dodecahydro-1*H*,12a*H*-cyclopenta[1,2]-phenanthro[7,8-b]indole (**20**) (Figure 10), after oxidation with chromic anhydride in acetone at 273 K, in the presence of phenylhydrazine and acetic acid.

Tosylation of 4α-14α-dimethyl-5α-ergost-8-en-3β-ol, another terpene isolated from *E. officinarum* latex, Mazoir et al. [47] obtained by hemisynthesis 3β-tosyloxy-4α,14α-dimethyl-5α-ergost-8-en-24-one (**21**), which after oxidation originated the derivatives 3β-tosyloxy-4α,14α-dimethyl-5α-ergost-8-ene-7,24-dione (**22**) and 3β-tosyloxy-4α,14α-dimethyl-5α-ergost-8-ene-7,11,24-trione (**23**) (Figure 10). Oxidation of eupho-lanost-8,24-dien-3β-ol, another metabolite found in *E. officinarum* latex, with the system sodium periodate-ruthenium (III) chloride trihydrate (NaIO_4_-(RuCl_3_,3H_2_O)) followed by esterification and acetylation reactions, originated (3*S*,5*S*,10*S*,13*S*,14*S*,17*S*)3β-acetyl-25,26,27-trisnorlanost-8-en-24-oate (**24**) [39] (Figure 10).

Beyond the production of triterpenoid derivatives obtained by hemisynthesis from triterpenoids isolated from the latex of *E. officinarum*, the same Moroccan team and others also from Morocco started to test the biological properties of the derivatives obtained or new derivatives along with the determination of their respective biological activities, with the purpose of obtaining compounds with better biological activities than the natural triterpenoids. This will fill the gap in studies on biological attributes of the natural triterpenes from *E. officinarum* latex or their derivatives—a gap that exists despite their known interesting pharmacological properties, including anti-inflammatory, antimicrobial and antiplasmodial activities [51].

With modifications on positions 3,7, 11 and 24 of obtusifoliol and 4α,14α-dimethyl-5α-cholest-8-en-3β-ol, isolated from *E. officinarum* latex, Mazoir et al. [52] obtained ten derivatives and evaluated the antifeedant effect on several insect species (*Spodoptera littoralis*, *Myzus persicae* and *Rhopalosiphum padi*), toxic effects on insect Sf9 and mammalian CHO cells, and phytotoxic effects on *Latuca sativa*. Out of the tested compounds, 4 had been already obtained: 3β-tosyloxy-4α,14α-dimethyl-5α-ergost-8-en-24-one (**21**), 4α,14α-dimethyl-5α-ergost-8,24-dien-3-one (**4**), 3β-acetoxy-4α,14α-dimethyl-5α-cholest-8-ene-7,11-dione (**17**), and 3β-tosyloxy-4α,14α-dimethyl-5α-cholest-8-en-7,11-dione (**19**). The remaining six compounds were β-tosyloxy-4α,14α-dimethyl-5α-cholest-8-ene (**25**), 4α,14α-dimethyl-5α-ergost-8-en-3,24-dione (**26**), 4α,14α-dimethyl-5α-cholest-8-en-3,11-dione-7-thiosemicarbazone (**27**), 4α,14α-dimethyl-5α-cholest-8-ene-3,11-dione-7-thiadiazoline (**28**), 4α,14α-dimethyl-5α-cholesta-7,9-diene-3-thiosemicarba-zone (**29**), and 4α,14α-dimethyl-5α-cholest-8-ene-7,11-dione-3-thiadiazoline (**30**) (Figure 10). The compounds 4α,14α-dimethyl-5α-cholest-8-en-3β-ol [9], 3β-tosyloxy-4α,14α-dimethyl-5α-cholest-8-ene-7,11-dione [19], 4α,14α-dimethyl-5α-ergost-8-en-3,24-dione (**26**) and 4α,14α-dimethyl-5α-cholest-8-ene-3,11-dione-7-thiosemicarbazone (**27**) were active in relation to *Myzus persicae*; 4α,14α-dimethyl-5α-cholest-8-en-3β-ol (**9**), 3β-acetoxy-4α,14α-dimethyl-5α-cholest-8-ene-7,11-dione (**17**), and 4α,14α-dimethyl-5α-ergost-8,24-dien-3-one (**4**) were active in relation to *Rhopalosiphum padi*; higher number of compounds were active in relation to *Spodoptera littoralis*, affecting insect growth, the C-3 substituent (C-3 hydroxyl is not essential for the insect growth) and C-7 substituent [52] being important. In addition, Mazoir et al. [52] also observed that the insect cells Sf9 were more sensitive to these 10 terpene derivatives than mammalian CHO cells, which could be explained by the differences in membrane composition and/or receptor affinity between insect and mammalian cells [52]. All of these derivative compounds had non selective moderate phytotoxic effects on radicle elongation of *Lactuca sativa*. The in vitro activity on *Leishmania infantum* promastigotes and *Trypanosoma cruzi* epimastigotes of these terpenoid derivatives was also evaluated by the same Moroccan team [53]. The choices made by the authors relied on the fact that Leishmaniosis and Chagas’ disease are still major worldwide health problems, with some medicines being ineffective or, in some cases, producing important side effects. The activities found for all compounds were moderate on both parasites, although some of them showed better activities. Thus, and in descending order of activity, the following compounds stand out: 4α,14α-dimethyl-5α-cholest-8-ene-3,11-dione-7-thiadiazoline (**28**), 3β-acetoxy-4α,14α -dimethyl-5α-cholest-8-ene-7,11-dione (**17**), 4α,14α-dimethyl-5α-ergost-8-en-3,24-dione-(**26**), 4α,14α-dimethyl-5α-ergosta-8,24-dien-3-one (**4**), and 4α,14α-dimethyl-5α-cholest-8-ene-3,11-dione-7-thiosemicarbazone (**27**). All of them had ED_50_ values (the effective dose to give 50% cell viability) lower than 10 μg/mL. The activity of the terpene derivatives on *Trypanosoma cruzi* was less effective. Only 4α,14α-dimethyl-5α-ergost-8-en-3,24-dione (**26**), 4α,14α-dimethyl-5α-cholest-8-ene-3,11-dione-7-thiosemicarbazone (**27**) and 4α,14α-dimethyl-5α-cholest-8-ene-3,11-dione-7-thiadiazoline (**28**) had ED_50_ values lower than 10 μg/mL [53]. The activities of these terpene derivatives on *L. infantum* and *T. cruzi* were associated with low to moderate effects on mammalian CHO cells, revealing a desirable selective toxicity [53]. The anti-parasite activities were higher in the tetracyclic triterpenes highly oxygenated with ketone/OH substituents at C-3 and C-7 and or C-11 and/or the presence of a substituent epoxy- or ketone group at C-24 in the lateral chain [53].

4α,14α-Dimethyl-5α-cholest-8-ene-3,11-dione-7-thiosemicarbazone (**27**) had moderate antileishmanial and antitrypanosomal activity [44]. Later, Mazoir and Benharref [54] obtained new thiosemicarbazone derivatives by hemisynthesis, treating hemisynthesized mono-, di-, and tricarbonyl compounds [e.g., 4α,14α-dimethyl-5α-ergost-8,24-dien-3-one (**4**), 4α,14α-dimethyl-5α-ergosta-8-en-3-one (**5**), (4*S*,5*S*,10*S*,13*R*,14*R*,17*R*)-8α,9α-epoxy-4α,14α-dimethyl-5α-cholestan-3-one (**13**), 3β-tosyloxy-4α,14α-dimethyl-5α-cholest-8-ene-7,11-dione (**19**), 3-acetoxy-30-nor-20-oxolupane (**31**), and 4α,14α-l-5α-cholest-8-ene-3,7,11-trione (**32**)] (Figure 10) from latex of Moroccan *E. officinarum*, with thiosemicarbazide and oxidation by chromic anhydride, with the purpose of finding new thiosemicarbazones derivatives with good yield and high regioselectivity. The same hemisynthesized mono-, di-, and tricarbonyl compounds were used for obtaining thiadiazolines since they possess several biological properties [53,55]. The new thiosemicarbazone derivatives obtained were 4α,14α-dimethyl-5α-ergost-8,24-dien-3-one thiosemicarbazone (**33**), 4α,14α-dimethyl-5α-cholest-8-en-3-one thiosemicarbazone (**34**), 3β-acetoxy-28-norlup-20-one thiosemicarbazone (**35**), 3β-tosyloxy-4α,14α-dimethyl-5α-cholest-8-ene-7,11-dione-7-thiosemicarbazone (**36**), and 4α,14α-dimethyl-5α-cholest-8-ene-3,7,11-trione-7-thiosemicarbazone (**37**) (Figure 10) [54]. 4α,14α-Dimethyl-5α-cholesta-7,9-dien-3-one thiosemicarbazone (**29**) was reported as a new compound, nevertheless, Mazoir et al. [52] had already obtained this triterpenic compound. Concerning the thiadiazoline derivatives, the compounds obtained were 4α,14α-dimethyl-5α-ergost-8,24-dien-3-one thiadiazoline (**38**), 4α,14α-dimethyl-5α-cholest-8-en-3-one thiadiazoline (**39**), 4α,14α-dimethyl-5α-cholest-7,9-diene-3-one thiadiazoline (**40**), 3β-aceto-xy-28-norlup-20-one thiadiazoline (**41**), 3β-tosyloxy-4α,14α-dimethyl-5α-cholest-8-ene-7,11-dione-7-thiadiazoline (**42**), and 4α,14α-dimethyl-5α-cholest-8-ene-3,7,11-trione-7-thiadiazoline (**43**) (Figure 10) [55].

Triterpene derivatives of *E. officinarum* latex have demonstrated toxic effects on two protozoan species *L. infantum* and *T. cruzi* [53], antifeedant and toxic effects on *S. littoralis*, an important crop pest, and selective cytotoxicity on insect and mammalian cells [52], as aforementioned. Bailen et al. [56] continued this work, studying the same biological properties previously reported [52,53] but hemisynthesizing new triterpene derivatives from obtusifoliol and 4α,14α-dimethyl-5α-cholest-8-en-3β-ol (**9**), major latex components of *E. officinarum* from the semi-arid regions of Morocco. From 4α,14α-dimethyl-5α-cholest-8-en-3β-ol (**9**), Bailen et al. [56] have obtained 14 derivatives, 4 being already hemisynthetized. 4α,14α-dimethyl-5α-cholest-8-ene-3,7,11-trione (**32**), (4*S*,5*S*,10*S*,13*R*,14*R*,17*R*)-8α,9α-epoxy-4α,14α-dimethyl-5α-cholestan-3-one (**13**), 8α,9α-epoxy-4α,14α-dimethyl-5α-cholestan-3β-ol (**14**) and 4α,14α-dimethyl-5α-cholest-8-en-3-one (**5**). 4α,14α-Dimethyl-5α-cholest-7,9-dien-3β-ol (**44**), 3-chloro-4α,14α-dimethyl-5α-cholest-8-en-7-one (**45**), 2-carbomethoxy-4α,14α-dimethyl-5α-cholest-2,8-dien-3-ol (**46**), 4α,14α-dimethyl-5α-cholest-8-en-3-one (**47**), 4α,14α-dimethyl-7-oxo-5α-cholest-8-en-3,4-lactone (**48**), 4α,14α-dimethyl-7,11-dioxo-5α-cholest-8-en-3,4-lactone (**49**), 8α,9α-epoxy-4α,14α-dimethyl-5α-cholest-3,4-lactone (**50**), 4α,14α-dimethyl-5α-cholest-7,9-dien-3,4-lactone (**51**), 4α,14α-dimethyl-3,4-seco-5α-cholest-7,9-dien-3,4-diol (**52**), 3-carbomethoxy-4-hydroxy-4α,14α-dimethyl-3,4-seco-5α-cholest-7,9-diene (**53**) were new derivatives (Figure 10). 4α,14α-dimethyl-5α-cholest-7,9-dien-3β-ol (**44**), 3-chloro-4α,14α-dimethyl-5α-cholest-8-en-7-one (**45**) and (4*S*,5*S*,10*S*,13*R*,14*R*,17*R*)-8α,9α-epoxy-4α,14α-dimethyl-5α-cholestan-3-one (**13**) were selective toxicants being most effective against *Leishmania*, although 3-carbomethoxy-4-hydroxy-4α,14α-dimethyl-3,4-*seco*-5α-cholest-7,9-diene (**53**) was the strongest antiparasitic with activity levels similar to or better than the reference drugs against *L. infantum* and *T. cruzi*, respectively. (4*S*,5*S*,10*S*,13*R*,14*R*,17*R*)-8α,9α-Epoxy-4α,14α-dimethyl-5α-cholestan-3-one (**13**), 3-chloro -4α,14α-dimethyl-5α-cholest-8-en-7-one (**45**). 4α,14α-dimethyl-7-oxo-5α-cholest-8-en-3,4-lactone (**48**) were not cytotoxic to mammalian CHO cells, which showed that they were selective to the parasites and therefore could be considered molecular leads for selective insecticides [56]. Compounds 3-carbomethoxy-4-hydroxy-4α,14α-dimethyl-3,4-*seco*-5α-cholest-7,9-diene (**53**) and 8α,9α-epoxy-4α,14α-dimethyl-5α-cholestan-3β-ol (**14**) had the strongest cytotoxic effects on insect-derived Sf9 cells. The compounds with an epoxide group such as (4*S*,5*S*,10*S*,13*R*,14*R*,17*R*)-8α,9α-epoxy-4α,14α-dimethyl-5α-cholestan-3-one (**13**), 8α,9α-epoxy-4α,14α-dimethyl-5α-cholestan-3β-ol (**14**), 8α,9α-epoxy-4α,14α-dimethyl-5α-cholesta-3,4-lactone (**50**), and 8α,9α,24,28-diepoxy-4α,14α-dimethyl-5α-ergost-3,4-lactone (**54**) had selective cytotoxic effects on Sf9 cells compared with mammalian CHO cells. 8α,9α,24,28-Diepoxy-4α,14α-dimethyl-5α-ergosta-3,4-lactone (**54**) as well as 8α,9α,24,28-diepoxy-4α,14α-dimethyl-5α-ergost-3β-ol (**55**) were hemisynthesized from obtusifoliol [56].

Epoxidation of the double bonds and hydroxylations of non-activated C–H groups of semisynthetic functionalized triterpenes 4α,14-dimethyl-5α,8α-8,9-epoxy-chole-stan-3β-yl acetate (**56**); 4α,14-dimethyl-5α-cholest-8-ene-3,7,11-trione (**32**); 4α,14-dimethyl-5α-cholesta-7,9-dien-3-one (**57**) and 4α,14-dimethyl-5α-cholest-8-en-3β-yl acetate (**58**), previously prepared from 4α,14α-dimethyl-5α-cholest-8-en-3β-ol (a natural insecticide present in *E. officinarum* latex) were performed by Mazoir et al. [57] with the purpose of obtaining optimized derivatives with high region selectivity and insecticidal activity. Several approaches had already been followed by this team and aforementioned. In the work presented in 2020, Mazoir et al. [57] used as reagents hydrogen peroxide (H_2_O_2_) and iodosobenzene (PhIO) catalyzed by porphyrin complexes (cytochrome P-450 models). Under these conditions, the compounds obtained were: 25-hydroxy-4α,14-dimethyl-5α-cholest-7,9-dien-3β-yl acetate (**59**), 25-hydroxy-4α,14-dimethyl-5α-cholest-8-ene3,7,11-trione (**60**), 4α,14-dimethyl-5α,7β-7,8-epoxychol-est-9-en-3-one (**61**), 8-hydroxy-4α,14-dimethyl-5α-cholest-9-ene-3,7-dione (**62**), 12α-hydroxy-4α,14-dimethyl-5α,7β-7,8-epoxycholest-9-en-3-one (**63**), and 4α,14-dimethyl-5α,8α-8,9-epoxycholestan-3β-yl acetate (**64**). The antifeedant and post-ingestive effects of these terpenoid derivatives were investigated for the insects *M. persicae*, *R. padi* and *S. littoralis* and Mazoir et al. [57] concluded that none of the compounds tested had significant antifeedant effects. All were more effective post-ingestive toxicants on *S. littoralis* larvae than the natural 4α,14α-dimethyl-5α-cholest-8-en-3β-ol (**9**), with 4α,14-dimethyl-5α,8α-8,9-epoxychole-stan-3β-yl acetate (**64**) being the most active. The authors also concluded that overall, the substituents at C-3 and C-7/C-8 modulated the insecticidal activity of the derivatives: acetylation at C-3/epoxidation at C-8 gave the maximum insecticidal effect (**56**), followed by carbonyl groups at C-3/C-7 with additional hydroxy groups (C-8, C-25) (**60**, **62**) or C-3 carbonyl and C-7 epoxide with C-8 unsaturation (**61**, **63**). The most active IGRs (insect growth regulators) had a C-3 carbonyl group and a C-8 epoxide or C-3 β-OH/C-7 unsaturation [56,57].

Beyond the insecticidal and antiparasitic activities aforementioned for some terpene derivatives obtained after hemisynthesis of terpenes isolated from *E. officinarum* latex, other biological activities have been reported. For example, Smaili et al. [58] reported that 3β-acetoxy-norlup-20-one (**31**) and 3-chloro-4α,14α-dimethyl-5α-cholest-8-ene (**64**), obtained by hemisynthesis from lupeol acetate (**2**) and 4α,14α-dimethyl-5α-cholest-8-en-3β-ol (**9**), respectively, were able in vitro to greatly reduce conidia formation and germination of *Verticillium dahlia*, and *Fusarium oxysporum* fsp. *melonis*, both causal agents of wilt, and *Penicillium expansum*, which is responsible for post-harvest rot that infects diverse fruits (e.g., tomato and apple). Such results indicate that those two derivatives act as fungistatic compounds [58]. The antibacterial activities of these derivatives were also evaluated in vitro. The phytopathogenic bacteria used were *Agrobacterium tumefaciens*, causal agent of crown gall disease, *P. syringae* pv. *tabaci* and *P. syringae* pv. *syringae*, which cause wild fire disease of tobacco, and diseases of various monocot and dicot plants, respectively. The results showed that compound 3-chloro-4α,14α-dimethyl-5α-cholest-8-ene (**64**) was more effective in inhibiting the growth of *P. syringae* pv. *tabaci* and *P. syringae* pv. *syringae*, even being similar to the positive control used (chloramphenicol). Concerning *Erwinia amylovora*, the causal agent of fire blight disease of pear and apple trees, 3-chloro-4α,14α-dimethyl-5α-cholest-8-ene (**64**) was also the only one of these compounds that showed antibacterial activity but at a moderate level and significantly lower than that recorded with the positive control [58].

Since 3β-acetoxy-norlup-20-one (**31**) and 3-chloro-4α,14α-dimethyl-5α-cholest-8-ene (**64**) were able to reduce in vitro conidia formation and germination of *Verticillium dahlia*, one of the most important vascular diseases reported on tomato plants, Smaili et al. [59] studied the effect of two triterpene derivatives [4α,14α-dimethyl-5α-cholest-7,9-dien-3β-ol (**44**) and 3β-tosyloxy4α,14α-dimethyl-5α-cholest-7,9-diene (**65**)], obtained after oxidation of 4α,14α-dimethyl-5α-cholest-8-en-3β-ol (**9**) isolated from *E. officinarum* latex, on the protection of tomato plants against *V. dahliae* in a greenhouse as well as in tomato plants derived from seeds that germinated in the presence of low concentrations of those two triterpenic derivatives. The results showed that they were able to significantly reduce disease severity at 10 μg/mL (e.g., reduction of leaf alteration index and of stunting index ranged from 52 to 68% and from 43 to 67%, respectively, while vessel discoloration was reduced by at least 95%) [59]. Moreover, the compounds were also able to elicit H_2_O_2_ accumulation before and after fungal inoculation, and enhance peroxidase and polyphenol oxidase activities. According to Smaili et al. [59], induction of protection against plant diseases by triterpenes of plant origin was reported for the first time.

Previously, Smaili et al. [58] concluded that 3-chloro-4α,14α-dimethyl-5α-cholest-8-ene (**64**) was effective in inhibiting the growth of *P. syringae* pv. *tabaci* in in vitro studies. Later, Smaili et al. [60] treated seeds of *Nicotiana benthamiana* with three hemisynthetic triterpenes, including 3β-acetoxy-norlup-20-one (**31**) and 3-chloro-4α,14α-dimethyl-5α-cholest-8-ene (**64**) derived from the latex of *E. officinarum,* in order to evaluate their ability to enhance resistance to *P. syringae* pv. *tabaci*. Smaili et al. [60] observed that soaking seeds in the triterpene derivatives did not harm germination and significantly reduced the diameter of the lesions in inoculated leaves, when compared to the control; bacterial growth was also significantly reduced in plants previously treated with the triterpenic derivatives by at least 0.54 logarithmic units when compared to the control. At the same time, the mock-inoculated leaves of plants that germinated in the presence of the triterpenic derivatives showed enhanced ascorbate peroxidase and catalase activities (two antioxidant enzymes). An increase of guaiacol peroxidase and polyphenol oxidase inoculated plants with *P. syringae* pv. *tabaci* was observed when pre-treated with the triterpenic derivatives.

Continuing the study on the effect of some triterpene derivatives obtained from triterpene isolated from *E. officinarum* latex, Smaili et al. [61] evaluated the effect of direct application of the 3β-acetoxy-norlup-20-one (**31**) and 3-chloro-4α,14α-dimethyl-5α-cholest-8-ene (**64**) on the growth of tomato seedlings under normal conditions or in the presence of the pathogens *V. dahliae* and *Agrobacterium tumefaciens*. After foliage spraying with these two derivatives, the authors observed a significant improvement of growth rate, fresh weight, dry weight and leaf area, an increased content of chlorophylls a and b, carotenoids, proline, and the activity of nitrate reductase (an enzyme which is correlated with growth and plant yield) [61]. In the presence of the infection by *V. dahliae*, triterpene derivatives reduced leaf alteration indexes induced by *V. dahliae*, particularly for 3-chloro-4α,14α-dimethyl-5α-cholest-8-ene (**64**). The browning index of the vessels caused by this phytopathogen was also much reduced, with the percentage of protection being 97–99% [61]. The diameter of lesions caused by the oncogenic strain C58 of *A. tumefaciens* was also reduced when pre-treated with those triterpenic derivatives. Moreover, these compounds also induced H_2_O_2_ accumulation and increased the activity of several antioxidant enzymes such as catalase, ascorbate peroxidase, and guaiacol peroxidase [61]. According to these results, Smaili et al. [61] concluded that the two derivatives are able to mediate resistance of tomato plant against bacterial and fungal diseases through improvement of antioxidant defences.

So far, diverse hemisynthesized triterpenoids obtained from isolated triterpenes of *E. officinarum* latex have been demonstrated to possess insecticidal and antimicrobial activities. Daoui et al. [48] have as purpose the improvement of these biological activities using in silico studies based on 3D molecular modelling techniques applied on 27 semisynthetic triterpene derivatives obtained from triterpenes isolated from *E. officinarum* and *E. resinifera* latices. To achieve this objective, Daoui et al. [48] have developed the three dimensional quantitative structure property relationships (3D-QSAR) based on Comparative Molecular Similarity Indices Analysis (CoMSIA) and Comparative Molecular Field Analysis (CoMFA) techniques. Such an approach enabled the authors to design 38 new derivatives and also to predict their pLD_50_ (log_10_[1/LD_50_]), where LD_50_ is the amount of sample which causes the death of 50% of the living beings of a group. Studies taking into account the absorption, distribution, metabolism, excretion (ADME), and toxicity (ADME-Tox) of the designed molecules led the authors to select four molecules as promising antibacterial and insecticidal molecules. The molecular docking test predicting the referential interactions that occur between the molecular structures and the receptors made it possible to find 3 molecules (**66**), (**67**), (**68**) (Figure 10) that were able to inhibit the MurE (PDB code: 1E8C) and EcR (PDB code: 1R20) proteins involved in the process of antibacterial and insecticidal activities and had greater stability than the reference molecule 24-methylen-elemo-lanosta-2,8,24-trien-7,11-dione (**69**) inside the MurE and EcR receptors pocket. The reference used was a triterpene derivative obtained through chemical modifications of the major component of *E. resinifera*, α-euphorbol (**70**) [53]. According to Daoui et al. [48], such observations may permit adoption of these molecules as references for the synthesis of insecticidal and antimicrobial activities.

## 5. Extracts and Bee Products from *Euphorbia* Origin

Although *E. officinarum* is used in folk medicine as antidiabetic or in the treatment of diseases of the respiratory and circulatory systems, pyelonephritis, treatment of wounds, skin infections and abscesses, headache, paralysis, apoplexy, among other ailments (see Introduction), very few works can be found that confirm these abilities. In fact, only the antimicrobial and insecticidal activities of triterpene derivatives from *E. offcinarum* latex have been extensively evaluated (see previous Section). Nevertheless, some works start to approach other attributes of *E. officinarum* extracts or their monofloral honeys. For example, El-Hawary et al. [33] tested the cytotoxic potential of fifteen *Euphorbia* species and concluded that methanolic extracts of the aerial parts of *E. officinarum* presented the highest activity against human colon adenocarcinoma (CACO2) cell line (IC_50_ = 7.2 μM). Chemical analysis through liquid chromatography-high resolution electrospray ionization mass spectrometry (LC-HR-ESIMS) and dereplication strategies using the Dictionary of Natural Products (DNP) database, followed by chemotaxonomic filtration, resulted in the characterization of 44 natural compounds from the 15 *Euphorbia* studied. For *E. officinarum*, two compounds were characterized but without activity (3β,7α-dihydroxy-4α,14α-dimethyl-5α-cholest-8-en-11-one (**10**) (Figure 2) and 8-methoxyingol 7,12-diacetate 3-phenylacetate (**3**) (Figure 3). After an orthogonal partial least square discrimination analysis (OPLS-DA), the authors concluded that the metabolite highly correlated with the CACO2 cytotoxicity was at *m*/*z* [M]^+^ 281.272 (time retention = 29.16), and was not yet isolated and identified in this species.

Beyond the antimicrobial and insecticidal activities of the derivatives obtained from natural triterpenes of the *E. officinarum* latex, Daoubi et al. [2] demonstrated that 8-methoxyingol 7,12-diacetate 3-phenylacetate (**3**) (Figure 3) was able to induce both G_0_/G_1_ cell-cycle arrest and human immunodeficiency virus type 1-long terminal repeat (HIV-1-LTR) promoter activation in a concentration-dependent manner, in the leukaemia cell line Jurkat-LTR-green fluorescent protein (Jurkat-LTR-GFR). According to these results, Daoubi et al. [2] suggested that 8-methoxyingol 7,12-diacetate 3-phenylacetate may be important for the development of therapies against HIV-latency. In fact, to eradicate HIV-1 has been difficult since the virus can be in reservoirs of latently infected cells and within them, the proviral DNA is integrated in the host’s genome but it does not actively replicate. In such a situation, the virus remains invisible to the host immune system and is not affected by antiviral drugs [62].

**Figure 11:**
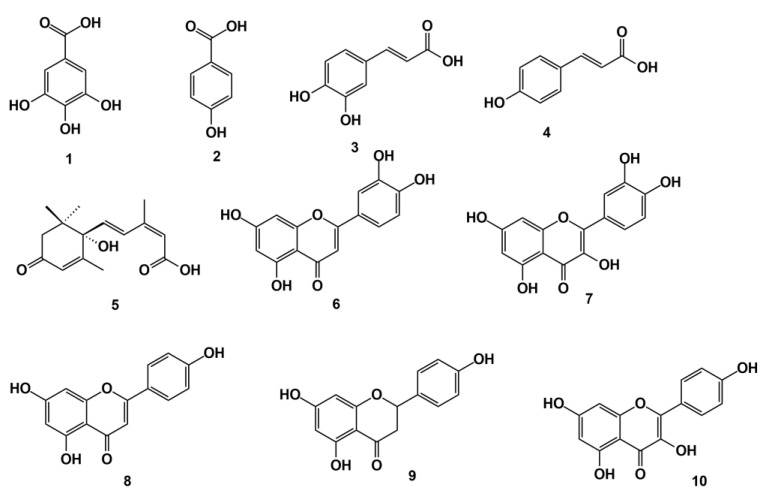
Phenols identified in the phenol fraction of *E. officinarum* honey from Morocco.

In the work developed by Boutoub et al. [63], they compared the antioxidant activity of aqueous plant extracts and monofloral honey of *E. officinarum* origin. They demonstrated that the ability of plant extracts for scavenging some free radicals such as 2,2-diphenyl-1-picrylhydrazyl (DPPH) and nitric oxide (NO) of plant extracts was more than 20 times greater than the activity of honey; likewise, the capacity for scavenging superoxide radical anions or antiacetylcholinesterase and anti-lipoxygenase were approximately 10 times higher in the plant extracts than in the honey samples. However,, the detailed chemical composition was not reported, only the total phenol, and in this case all aqueous extracts had higher amounts of these metabolites than the honey samples. The extraction conditions (time, temperature and plant solvent ratio) were determinant of the total concentration of phenols as well the antioxidant activity and inhibition of the α-glucosidase activity of the *E. officinarum* aqueous extracts [64]. The same team [65] also concluded that the activities found for honey samples could be attributed to the phenol fraction since the phenols extracts isolated from the monofloral honey of *E. officinarum* origin had better activities than the entire honey. In this case, Boutoub et al. [65] presented a preliminary chemical composition of the extracts (gallic acid (**1**), *p*-hydroxybenzoic acid (**2**), caffeic acid (**3**), *p*-coumaric acid (**4**), abscisic acid (**5**), luteolin (**6**), quercetin (**7**), apigenin (**8**), naringenin (**9**) and kaempferol (**10**)) (Figure 11) [65]. The authors [63,65] studied monofloral honey because in Morocco the *Euphorbia* honey is considered the most precious by the consumers but it has been scarcely studied, although the physico-chemical and palynological characteristics, generally needed for honey characterisation, have already been found [63,66,67,68,69]. Many times, beekeepers labelled the honey as being only of *Euphorbia* origin, although three honey types of this genus are produced (*E. officinarum* subsp. *echinus*, *E. resinifera* and *E. regis-jubae*) [48,70]. The work of Abderrahim et al. [71] demonstrated the antioxidant, synergistic antimicrobial and burn wound healing activities of monofloral honey of *Euphorbia* origin (without any specification of the species) mixed with *Allium sativum*. This mixture had higher wound healing activity, since shorter epithelialization and wound contraction time was observed, as well as better histological recovery of the treated tissues [71].

Another beeproduct is propolis, a plant-derived product that bees collect from resins and exudates from diverse parts of the plants, and subsequently transport to the hive, mixing it with beeswax. Bees use propolis to protect the hive against intruders and pathogenic microorganisms [72]. The detailed chemical composition and antimicrobial activity of propolis from a semi-arid region of Morocco were evaluated by Chimshirova et al. [72]. Fifteen compounds were isolated and identified, some of them being already reported as constituents of plants in the genus *Euphorbia*, particularly the macrocyclic diterpenes and triterpenoids, as well as other groups of known compounds (e.g., coumarins, phenolic acids) and new ones (e.g., 29-norlanost-3β-hydroxy-8-ene-7,11-dione). The macrocyclic diterpenes, particularly ingol diterpenes containing a phenylacetyl group were only found in the latex of *E. resinifera* and *E. officinarum*. However, the ingol diterpenes found in propolis of this work were those isomers characteristic of the *E. resinifera* latex. Such results may indicate the utilization of latex of *E. resinifera* by bees for making propolis, but also from *E. officinarum*, since obtusifoliol is generally present in the *E. officinarum* latex. *p*-hydroxybenzoic acid was reported in this propolis sample, being also observed in honey of *E. officinarum* and *E. resinifera* origins [65].

## 6. Conclusions

*Euphorbia officinarum* is geographically limited to Morocco, Western Sahara, Algeria and Mauritania. This species has been used in folk medicine in various ways: as anti-diabetic; in the treatment of skin diseases, although later it was concluded that the main treatment purpose was the elimination of helminths; when associated with other plants (*Opuntia ficus-barbarica*, *Zea mays* and *Ziziphus lotus*) and honey, in the treatment of pyelonephritis and cystitis. So far, fifteen compounds (diterpenes, triterpenes and sterols) have been isolated and identified in the *E. officinarum* latex from Morocco. The *E. officinarum* honey is considered the most precious; nevertheless, many times it is mixed with other Euphorbiaceae honeys. The chemical composition of the phenolic fraction of the monofloral honey was found to include ten compounds. More than seventy hemisynthesized compounds were obtained from some triterpenes of *E. officinarum* in order to obtain compounds with higher insecticide and antimicrobial activity. The in silico studies indicated that three hemisynthesized compounds were able to inhibit proteins involved in the process of antibacterial and insecticidal activities and also presented great stability inside the protein receptors pocket. They are, therefore, of interest for possible adoption as references for the synthesis of antibacterial drugs and insecticides.

This review shows an insufficiency of knowledge on the chemical composition of latex, flowers and other organs of *E. officinarum*, and the need for further investigation. Greater insight into the relationship between the chemical structures of the natural compounds or normalized extracts and biological properties is also needed. A possible relationship of the chemical composition of flowers and that of honey is another field requiring investigation. Since honey is appreciated by consumers and in order to increase the commercial value of a monofloral *E. officinarum* honey, it would be important to find one or more specific markers for this type of honey. Since many hemisynthesized compounds have been obtained, it is necessary to assay more biological activities of such compounds and not only antimicrobial and insecticidal activities.

## Data Availability

The data is contained within the manuscript.
